# Giant abdominal cyst in a young female patient: A case report

**DOI:** 10.1016/j.ijscr.2020.06.085

**Published:** 2020-06-24

**Authors:** Cláudia Leite, Bruno Barbosa, Natália Santos, Ana Oliveira, Carlos Casimiro

**Affiliations:** General Surgery, Centro Hospitalar Tondela-Viseu, 3504-509, Viseu, Portugal

**Keywords:** Abdominal cyst, Ovarian cyst, Abdominal distension, Adnexectomy, Mucinous cystadenoma, Case report

## Abstract

**Introduction:**

Most abdominal cysts derive from the ovary. The range of differential diagnoses is wide. Unfortunately, imaging studies not always determine its origin.

**Presentation of case:**

The authors present the case of a 20-year-old female patient, admitted to the emergency department due to abdominal pain and distension, whose imaging studies revealed a gigantic abdominal cyst of unknown origin. She underwent an exploratory laparotomy that disclosed an ovarian cyst that was removed by a left adnexectomy. It weighed 10Kg and was 60 cm wide. The pathology report showed a mucinous cyst adenoma.

**Discussion:**

Once a patient present with an abdominal cyst, one should always consider the extensive list of differential diagnoses. In premenopausal women, ovarian cysts are very frequent. Cysts may grow to considerable size. Our patient was symptomatic, malnourished and dehydrated. Neither ultrasonography nor computed tomography were able to define the origin of the cyst. Persistent ovarian cysts larger than 10 cm, particularly if symptomatic, should be considered for surgery.

**Conclusion:**

Progressive abdominal distension in premenopausal women should raise suspicion of an ovarian tumor, such as mucinous cystadenoma. These tumours are benign, but when their size is considerable, complications do arise and their surgical removal may be life threatening.

## Highlights

• We should consider the differential diagnoses of an abdominal cyst.
• In premenopausal women, ovarian cysts are frequent and may grow to considerable size.
• Some ovarian cysts cause symptoms, such as obstipation, vomiting and malnourishment.
• US is the primary imaging study for an ovarian cyst.
• Persistent simple ovarian cysts larger than 10 cm should be considered for surgery.

## 1 Introduction

Most abdominal cysts derive from the ovary, which may vary between simple and functional cysts to malignant neoplasms. The list of differential diagnoses is extensive and includes peritoneal cyst, para-ovarian cyst, appendiceal mucocele, cystic adenomyosis, liver, pancreatic or choledochal cyst, lymphocele, cystic lymphangioma, duplication intestinal cyst, bladder diverticulum, to name just a few.

Ovarian cysts may become symptomatic as their size increases, presenting with abdominal, pelvic or lumbar discomfort or pain, progressive abdominal distension, nausea or vomiting.

Unfortunately, imaging studies such as ultrasound (US), computed tomography (CT) and magnetic resonance (MRI), not always determine the origin of the cyst, thus limiting its diagnostic usefulness [1].

Indications for surgery include a symptomatic or rapid growing cyst, and when its malignant potential cannot be excluded.

This work is reported in line with the SCARE 2018 criteria [2].

## 2 Presentation of case

A 20-year-old female patient was admitted to our emergency department (ED) due to abdominal pain and distension, nausea, vomiting and constipation. Her mother died young with breast cancer.

Her skin and mucosae were pale and dehydrated. Breath sounds were diminished at the pulmonary bases. Her abdomen was significantly distended, with stretch marks and oedema of its wall, was depressible by palpation with diffuse tenderness, without guarding. Bowel sounds were diminished and percussion produced a dull note. Her limbs were thin. Blood tests were normal.

**Fig. 1:**
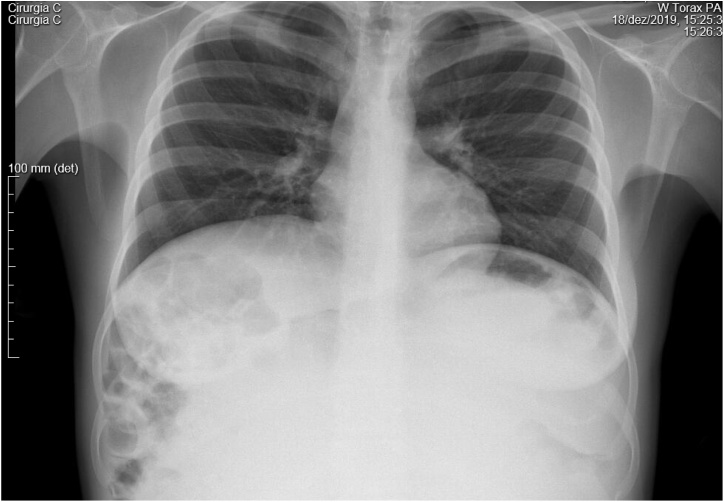
Thoracic X-ray showing symmetrical elevation of both diaphragmatic domes and presence of collapsed small bowel in the right upper abdominal quadrant.

**Fig. 2:**
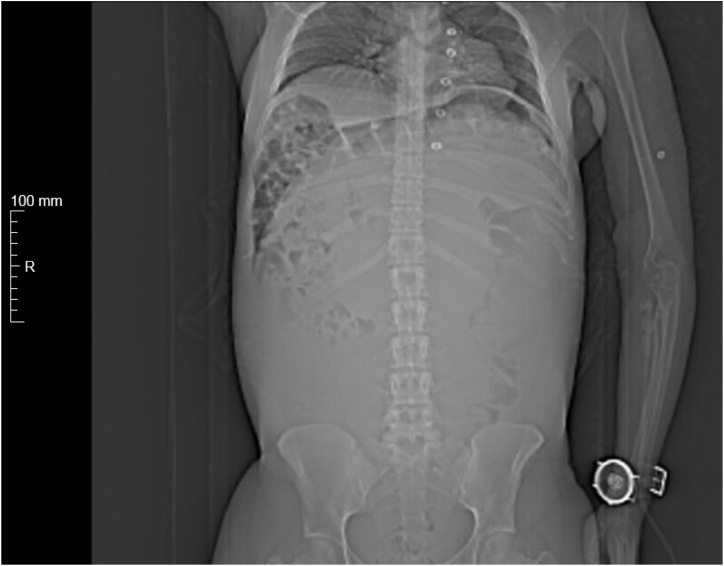
Thoracic, abdominal and pelvic CT showing symmetrical elevation of both diaphragmatic domes and presence of collapsed small bowel in the right upper abdominal quadrant.

**Fig. 3:**
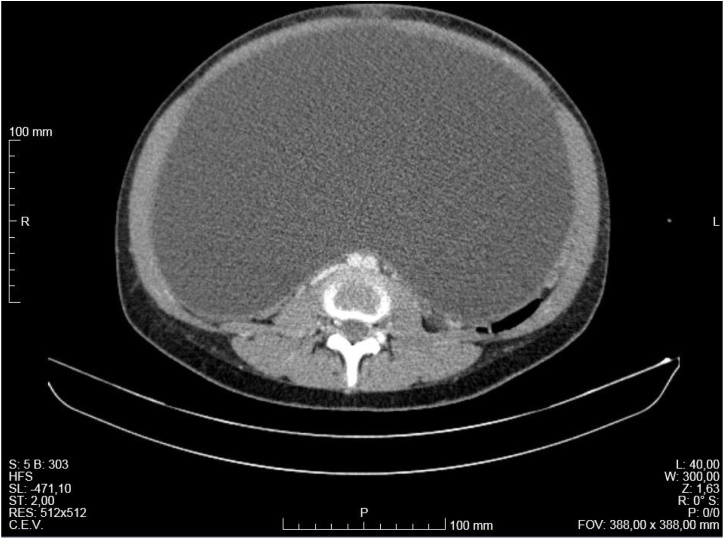
Axial image of abdominal and pelvic CT showing the large size of the cyst with a thin and regular wall, that occupied practically all the abdominal and pelvic cavities, with no contrast-enhanced solid areas, that deviated and collapsed the abdominal and pelvic viscera.

**Fig. 4:**
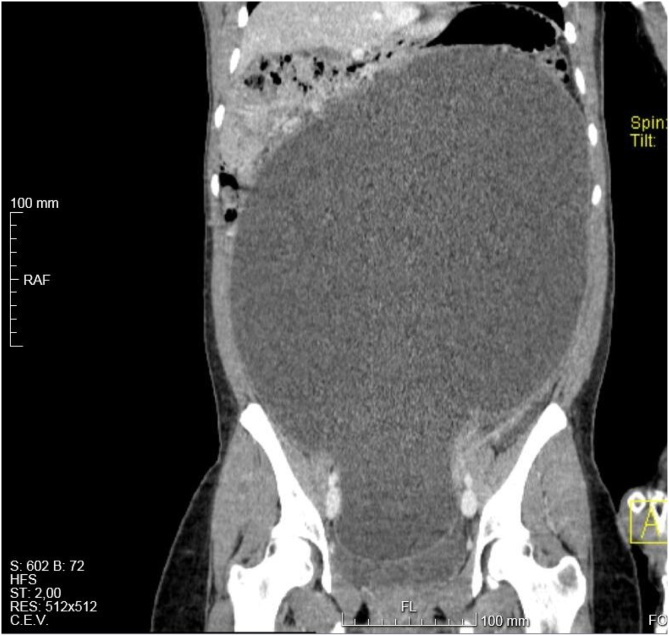
Coronal image of abdominal and pelvic CT showing the large size of the cyst and its effects on the surrounding structures.

**Fig. 5:**
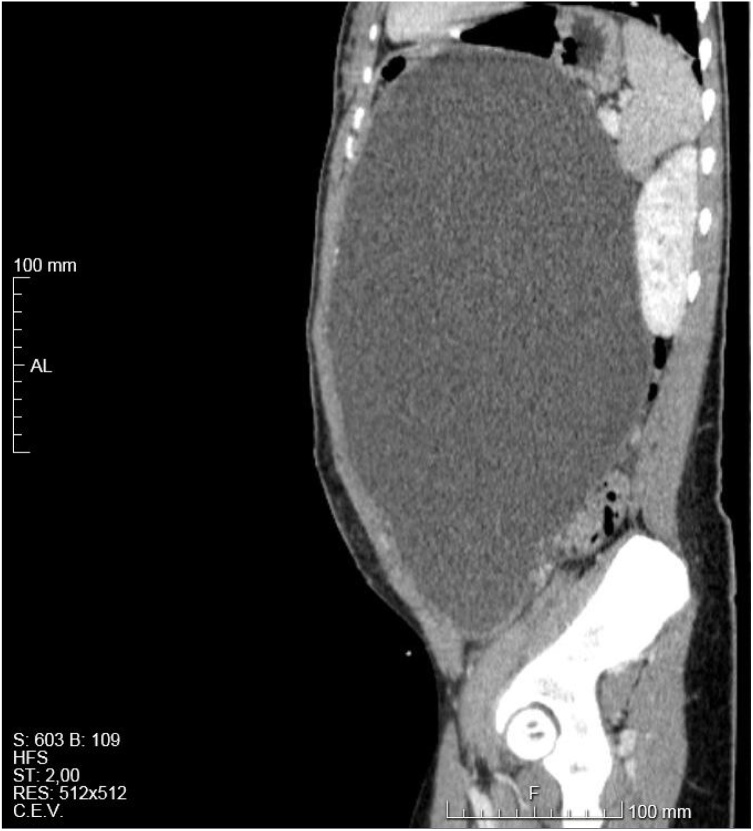
Sagittal image of abdominal and pelvic CT showing the large size of the cyst and its effects on the surrounding structures.

She had an abdominal and pelvic US that revealed a large abdominal cyst of unknown origin, followed by an X-ray and CT (Fig. 1, Fig. 2, Fig. 3, Fig. 4, Fig. 5) that showed an enormous cyst, still of unknown origin.

Tumour biomarkers alpha fetoprotein (AFP), carcinoembryonic antigen (CEA), cancer antigen 19.9 (CA19.9), cancer antigen 125 (CA 125) and human epididymal protein 4 (HE4) were normal. The risk of malignancy algorithm (ROMA) value was 4.1 %. Echinococcus granulosus serology test was negative.

**Fig. 6:**
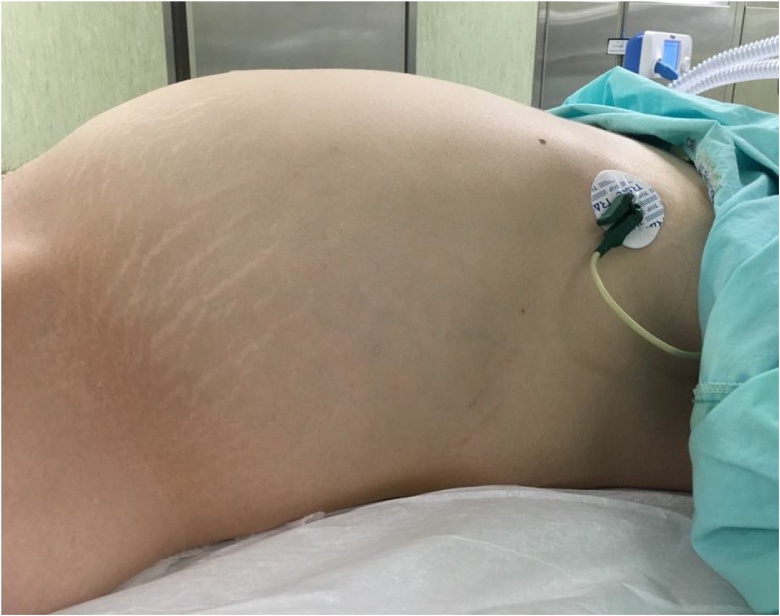
Photograph taken immediately before surgery showing a significant abdominal distension and multiple recent stretch marks.

**Fig. 7:**
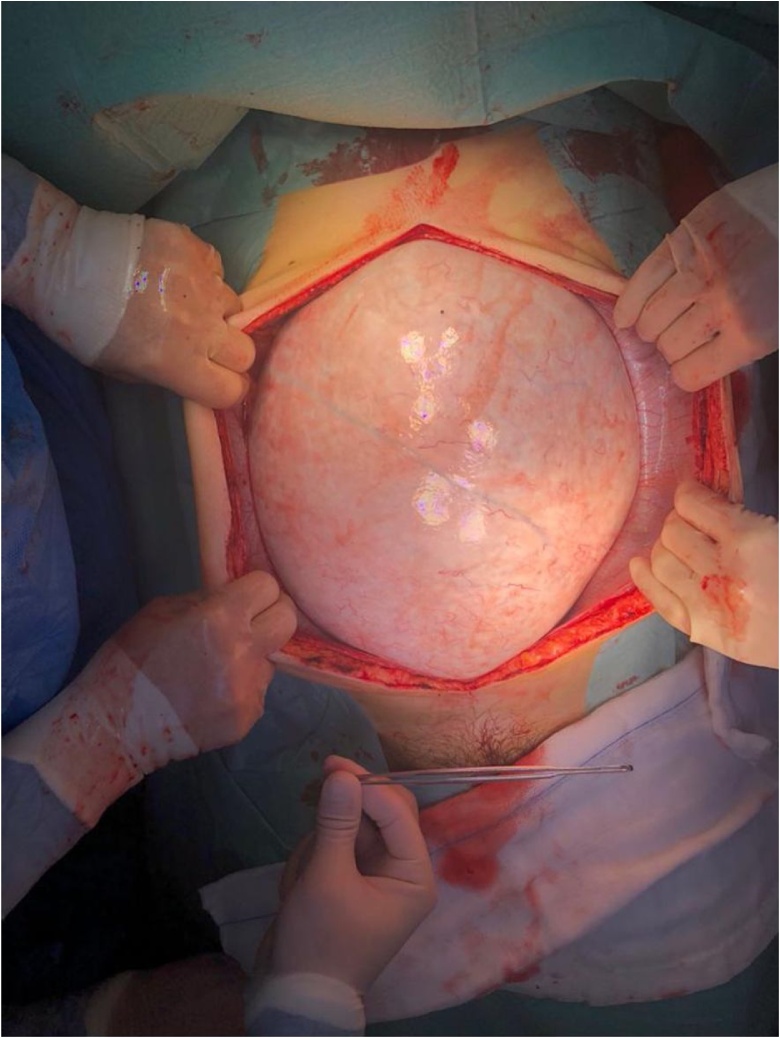
Photograph taken on entering the abdominal cavity. The cyst occupied all the peritoneal cavity and was 60 cm wide.

**Fig. 8:**
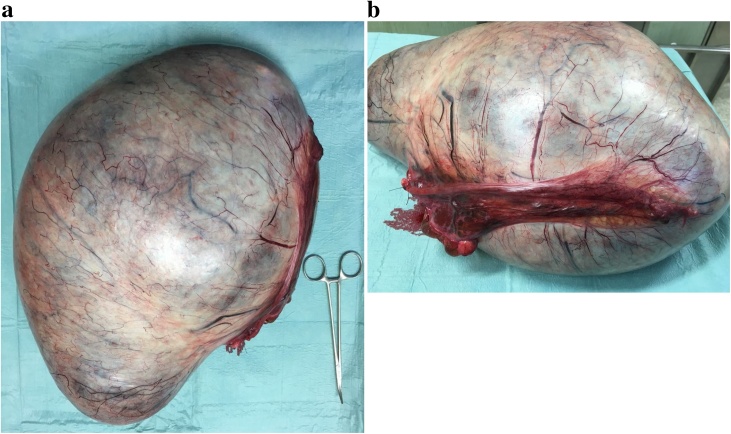
a) Photograph of the specimen of the left adnexectomy. Its serosa was bluish white, bright, smooth and highly vascularized. b) Photograph of the specimen of the left adnexectomy. The left uterine tube was 20 cm long.

Hence, she underwent an exploratory laparotomy (Fig. 6), and it was soon confirmed that the cyst had replaced the left ovary (Fig. 7). Therefore, a left adnexectomy became mandatory (Fig. 8). The procedure ran uneventfully. There were no ascites, peritoneal implants nor liver nodules. The small bowel was collapsed in its entire length and located in the sub-hepatic recess. The sigmoid colon was also collapsed and had a few soft adhesions to the cyst. The right ovary, the uterus and the appendix had no suspicious macroscopic appearance. There was no spillage nor perforation of the cyst. Pelvic peritoneal fluid was sent for cytology - no neoplastic cells were identified. The cyst weighted 10,2Kg.

She had an uneventful recovery and was discharged home on the 5th post-operative day.

**Fig. 9:**
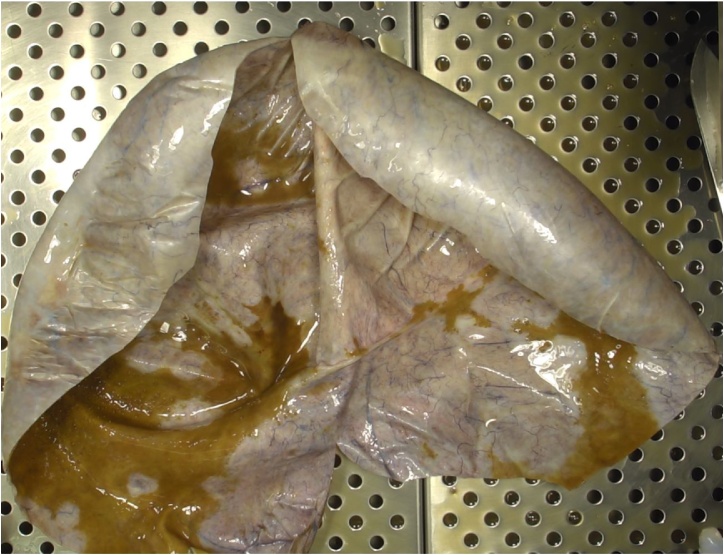
Photograph of the macroscopy of the specimen (opened).

The pathology report revealed an ovarian mucinous cystadenoma. The cyst had a thin wall (Fig. 9), internally coated by a single layer of a mucous secreting cylindrical epithelium of the endocervical type, with caliciform cells. There was no atypia, stratification nor mitotic activity. There was no residual normal ovarian tissue either.

She remains clinically asymptomatic.

## 3 Discussion

Once a patient present with an abdominal cyst, one should always consider the extensive list of differential diagnoses.

In premenopausal women, ovarian cysts are very frequent, and most are benign and functional. Cysts may grow to considerable size. Some cysts may be symptomatic, presenting with discomfort in the lower abdomen or severe pain from complications: torsion, rupture or haemorrhage [3,4]. Severe cases include hypotension or abdominal compartment syndrome. However, up to 88 % of ovarian cysts are asymptomatic [5]. Our patient was symptomatic, underweight, malnourished and dehydrated.

US is the primary imaging study for an ovarian cyst [3]. A normal ovary is 2.5−5 cm long, 1.5−3 cm wide and 0.6–1.5 cm thick. Simple cysts have a homogeneously thin and rounded wall, and an unilocular appearance that is either hypoechoic or anechoic. They generally measure 2.5−15 cm in diameter and a posterior acoustic enhancement (a hyperechoic area) may be noticeable deep to the fluid-filled cyst [4,5]. These cysts are unlikely to be malignant. Papillary projections in a cyst should raise the suspicion of malignancy [6]. Despite the size of the cyst of our patient, it was uniloculated and had a thin wall.

Transabdominal US is better than endo-vaginal US for assessing large cysts and their complications. It also allows assessment of other intra-abdominal structures. In our patient, it became utterly impossible to describe other structures besides her cyst.

Needle aspiration for cytology provides inaccurate results and owing to its associated complications (bowel perforation, cyst rupture, potential risk of peritoneal dissemination of malignant cells), it is not recommended. Its use has largely been supplanted by US [7].

Removing the cyst intact for histology is the gold standard and may require an oophorectomy [8]. It should always be obtained fluid from peritoneal washings for cytology. The left ovary of our patient had completely turned into that giant cyst. Thus, a left adnexectomy became mandatory.

CA125 is a protein expressed on the cell membrane of normal ovarian tissue and ovarian carcinomas. Its value, despite its low sensitivity and specificity, is raised in 85 % of patients with epithelial ovarian carcinomas [9]. HE4 is not expressed on normal ovarian cells but is highly expressed in ovarian cancer. It has a high specificity for the diagnosis of ovarian cancer. Both were normal in our patient.

CT is more sensitive yet less specific than US in detecting ovarian cysts [10]. It allows assessment of abdominal and retroperitoneal structures [11].

Still, neither US nor CT were able to define the origin of the cyst in our patient. MRI could have been a good option since it has better soft tissue contrast than do CT.

Persistent simple ovarian cysts larger than 10 cm, particularly if symptomatic, and complex ovarian cysts should be considered for surgery.

To the best of our knowledge, this is one of the largest ovarian cysts being reported in such a young age, accounting for, approximately, one-fifth of the weight of the patient.

## 4 Conclusion

Progressive abdominal distension in premenopausal women should raise suspicion of an ovarian tumour, such as mucinous cystadenoma. These tumours are benign, but when their size is considerable, complications do arise and their surgical removal may be life threatening.

## Declaration of Competing Interest

Nothing to declare.

## Funding

No funding.

## Ethical approval

Clinical case exempt from ethical approval in our institution.

## Consent

Written informed consent was obtained from the patient for publication of this case report and accompanying images. A copy of the written consent is available for review by the Editor-in-Chief of this journal on request.

## Author contribution

Cláudia Leite - data collection, data analysis and interpretation, writing the paper.

Bruno Barbosa - data collection, writing the paper.

Natália Santos - data collection, data analysis and interpretation.

Ana Oliveira - operated the patient, data analysis and interpretation, writing the paper, manuscript review.

Carlos Casimiro - manuscript review.

## Registration of research studies

Clinical case report, not formal research project.

## Guarantor

Cláudia Leite and Ana Oliveira.

## Provenance and peer review

Not commissioned, externally peer reviewed.
